# The Institutional Library

**Published:** 1915-02-06

**Authors:** 


### THE INSTITUTIONAL LIBRARY

### Mothercraft and Health Visitors.

The Infant: Nutrition and Management. By Eric Pritchard, M.A., M.D., M.R.C.P. (London: Edward Arnold. 1914. Price 3s. 6d. net.)

Lactation is surely one of those conditions to which justice has not been done by the rank and file of the medical profession. Its early phases pass almost unheeded by the obstetrician, or receive attention for a matter of days only, while, as likely as not, further advice is not sought until the child has been weaned and breast feeding complacently regarded as an inevitable failure. The problems associated with such an apparently simple matter as breast feeding are more numerous and more complex than the average health visitor, or, for the matter of that, the general medical practitioner, imagines. As regards the population at large, there has been effected a very satisfactory degree of enlightenment during the past decade concerning those matters which are included in the term "mothercraft." The results achieved by various workers, schools for mothers, infant consultations, and suchlike bodies are encouraging, and have, we may hope, scotched for ever the abysmal ignorance which prevailed formerly in certain communities. The management and feeding of the infant form a subject about which no author of any reputation will write without a considerable sense of the responsibility he is undertaking; there is so much chaff to be sifted from the grain that careful research and keen personal observation are necessary to elicit sound information or to promulgate methods and rules of substantial worth. Dr. Eric Pritchard is one of the keen workers for the reduction of infantile mortality and for the general improvement of the health of the child under one year old. The book which he has lately published is well worth reading; it may, indeed, be specially recommended to young medical men about to start in general practice. They will be unlucky if, sooner or later, they do not get a certain *kudos* through acting on some piece of practical information in this book. Dr. Pritchard, when necessary, does not hesitate to trample upon tradition, but he carefully exposes his case and argues for something better than that against which he inveighs.

The first half of the book deals with feeding and nutrition, and here the author is greatly helped by his large experience of test feeds and his extensive clinical work at the Queen's Hospital for Children, and at the St. Marylebone Infant Consultations. Dr. Pritchard should perhaps be congratulated on the success with which he has been able to induce mothers to carry out his instructions in many complicated cases. Breast-feeding is dealt with at length, and its difficulties are not underrated, nor its merits exaggerated. The much-advertised galactagogues receive a verdict of "no use," and the common accessories so strongly recommended by friends of the family are not encouraged. It is pointed out that infants get quite good supplies of milk from underfed mothers—a fact which is rarely believed, though often confirmed. Extra food for the mother has a distinct influence on the fat content of the milk, but little or no other effect, except, of course, on the nutrition of the mother. Dr. Pritchard lays stress on the fact that the digestive functions of the infant are only slowly developed, and that time must be allowed for the gastric juices to be educated to deal with foreign proteins; theoretically it is desirable to predigest cow's milk for twenty-four hours with pancreatic extract before giving it to a new-born infant, but practically the author concedes that six hours' predigestion is sufficient. He does not think that milk digested in this way is too nauseating for infant consumption, for new-born infants will swallow most liquids with complete indifference. The babe is to be gradually taught to digest the milk by reducing the time of pancreatisation each day, so that in thirty-six days it will be taking undigested milk.

That artificially fed babies are almost invariably overfed is a truism, the importance of which is not often sufficiently realised, and the author does well in drawing attention to the necessity of allowing only a restricted dietary to the ordinary slum infant. Nature is wiser than we are in providing such infants with a comparatively small allowance of breast milk, for the overclothing and the hot, ill-ventilated rooms with which these children have to put up deprive them of that stimulus which creates a demand for extra food by quickening the vital processes and raising tone. To give increased quantities of food before there is a physiological requirement is to court disaster. The degree of overfeeding in a slum infant becomes relatively greater in hot weather, and the danger it runs becomes correspondingly increased. Hence it is necessary to be on one's guard in hot weather lest the increased thirst of the infant be mistaken for hunger, and a second bottle be given because the first was drained dry and yet the child appeared unsatisfied. A useful warning may be quoted here: "To keep infants warm to prevent diarrhœa is as irrational as to confine infants in stiff binders to strengthen their backs." As the reader proceeds with the book he will be able further to judge the practical nature of the author's views and instructions, whether on the training of nerve centres in infants, on the prevention of constipation, or on the uses of dried milk. He justly and strongly condemns the almost wicked practice of clearing out the meconium from the new-born by the dose of castor-oil on which the midwife so often insists. To the question of the administration of petroleum the author devotes quite a long chapter. Enough has been said to recommend this book as a practical working treatise, and it may be allowable to carp at one small fault. Such a practical man as the author should surely not expect drops of tincture of opium to have any effect in relieving pain when instilled into the external auditory meatus.

### The "Legal" Engagement and Case Book.

The "Legal" Engagement and Case Book for Midwives and Maternity Nurses. By Sarah Macdonald. (London: The Scientific Press, Ltd. Price 1s.)

This case-book consists of a series of forms and charts to be used by maternity nurses in the course of their work. Each group includes a list of articles required by the mother and child respectively, a temperature chart, a form on which to indicate the progressive involution of the uterus, a weight chart, and last, but not least, an engagement bond to be signed by the patient and legalised by a sixpenny stamp. As various inquiries in our Bureau of Information have abundantly shown, nurses will be well advised to have this bond signed in every case, so that if any hitch occurs they "may sue for breach of contract," and may obtain "damages." The forms are obviously convenient and useful.

